# Palliative care in Mozambique: Physicians’ knowledge, attitudes and practices

**DOI:** 10.1371/journal.pone.0238023

**Published:** 2020-08-24

**Authors:** Emilia Pinto, Gustavo Marcos, Camila Walters, Ferraz Gonçalves, Jahit Sacarlal, Luisa Castro, Guilhermina Rego

**Affiliations:** 1 School of Medicine, Porto University, Porto, Portugal; 2 Pain Unit, Maputo Central Hospital, Maputo, Mozambique; 3 Anesthesiology Department, Maputo Central Hospital, Maputo, Mozambique; 4 School of Medicine, Universidade Eduardo Mondlane, Maputo, Mozambique; 5 Anesthesiology Department, Vanderbilt University, Nashville, Tennessee, United States of America; 6 Palliative Care Service, Portuguese Oncology Institute, Lisbon, Portugal; 7 Center for Health Technology and Services Research (CINTESIS), University of Porto, Porto, Portugal; Boston University School of Medicine, UNITED STATES

## Abstract

**Background:**

Palliative care is an essential part of medical practice but it remains limited, inaccessible, or even absent in low and middle income countries.

**Objectives:**

To evaluate the general knowledge, attitudes, and practices of Mozambican physicians on palliative care.

**Methods:**

A cross–sectional observational study was conducted between August 2018 and January 2019 in the 3 main hospitals of Mozambique, in addition to the only hospital with a standalone palliative care service. Data was collected from a self-administered survey directed to physicians in services with oncology patients.

**Results:**

Two hundred and seven out of 306 physicians surveyed answered the questionnaire. The median physician age was 38 years. Fifty-five percent were males, and 49.8% residents. The most common medical specialty was surgery with 26.1%. Eighty percent of physicians answered that palliative care should be provided to patients when no curative treatments are available; 87% believed that early integration of palliative care can improve patients’ quality of life; 73% regularly inform patients of a cancer diagnosis; 60% prefer to inform the diagnosis and prognosis to the family/caregivers. Fifty percent knew what a “do-not-resuscitate” order is, and 51% knew what palliative sedation is. Only 25% of the participants answered correctly all questions on palliative care general knowledge, and only 24% of the participants knew all answers about euthanasia.

**Conclusions:**

Mozambican physicians in the main hospitals of Mozambique have cursory knowledge about palliative care. Paternalism and the family-centered model are the most prevalent. More interventions and training of professionals are needed to improve palliative care knowledge and practice in the country.

## Background

Palliative care is an essential part of medical practice but it remains limited, inaccessible, or even absent in low and middle income countries (LMICs). There is often a limited number of specialized professionals, delayed detection of pathologies, and inaccessible and insufficient treatment options in these countries. Palliative care availability is an urgent need aggravated by increasing global oncologic prevalence, which is expected to double in 2 decades, with fifty percent of these cancers occurring in LMICs [1]. In a recent study of 48 African countries, 19% had no hospices or hospice services and only 22% had documented hospice polices, with rare exceptions such as Uganda, Kenya and South Africa. Few countries in Africa have organized palliative care systems [2].

According to the Joint United Nations Programme on HIV/AIDS (UNAIDS), Mozambique has the world´s third highest HIV prevalence at 13.2%. The World Alliance of Hospice and Palliative Care (WHPCA), states that most people living with HIV / AIDS who need such care are in Africa, with 78% of adult patients in LMICs. Forty-nine percent of children with palliative care needs in the whole world are in Africa [3].

Mozambique is one of the 11 African countries with palliative care policies, and one of the 6 African countries along with Rwanda, Swaziland, Tanzania, Zimbabwe, and Malawi, that provide a standalone palliative care service. However, in Mozambique, palliative care access is limited and not fully integrated into the health care system. There are knowledge gaps and undesirable attitudes by policymakers and healthcare professionals [4]. The African Palliative Care Association recommends palliative care content to be integrated in training curricula for health care workers in Mozambique [4] and it is important that it includes both theoretical knowledge and practical skills [5].

Few studies have been published about palliative care in Mozambique [2]. In this study we survey knowledge, attitudes and practices of Mozambique physicians in relation to palliative care philosophy, disclosure of diagnosis, breaking bad news, therapeutic approach, and end-of-life decisions. This is the first survey of its kind in Mozambique.

## Methods

A cross-sectional study was carried out between August 2018 and January 2019 in Mozambique at the major hospitals in the southern, central, and northern regions of the country—Maputo, Beira, and Nampula—as well as Xai-Xai Provincial Hospital (XXPH), the only hospital with a standalone palliative care service in Mozambique.

To ensure adequate accuracy in our proportion estimates, we compute the desired sample size for a 95% confidence interval, an accuracy of 0.05 and an expect proportion of 0.50, generating a sample size of 306 participants from a population of 868 physicians in the 4 hospitals: Maputo—553 (195), Beira– 163 (57), Nampula– 118 (42) and Xai-Xai– 34 (12) physicians.

The study was directed to physicians—general practitioners, residents and specialists—in the main hospital services with oncological patients: medicine, gynecology, surgery, urology, pain, oncology, dermatology, pediatrics, otorhinolaryngology, maxillofacial, surgery, gastrology, ophthalmology, emergency medicine, and orthopedics services. Surveys were distributed in paper and self-administered. The principal investigator and trained pain professionals distributed the surveys and informed consents, consecutively, to all physicians present at the services during the study period, willing to participate in the study. Physicians were informed about the study aims and the importance of their responses to the completion of the study. When available, physicians answered the surveys and the team collected the survey immediately, otherwise answered surveys were collected in the end of the shift.

The applied survey (Mozambique Palliative Care Knowledge and Attitudes Survey or MPCKAS) was the result of merging a modified questionnaire conducted in China [6] and another led in Portugal [7]. From the Chinese survey, we used questions related to demographic data, concept and philosophy of palliative care, disclosure of diagnosis, breaking bad news and end of life issues (Q1-Q12; Q18—Q29). From the Portuguese survey, we used questions complementary to the questions above for diagnosis information and treatment decision (Q13—Q17). By applying a blend of these 2 questionnaires we intended to relate physicians’s knowledge to their attitudes and practices. The pre-final survey, in Portuguese, was obtained from a process that includes translation and back-translation, performed by two professional translators and revised by a team of experts from the Portuguese Oncology Institute, according to published guidelines for translation and validation of surveys [8].

The pre-final version of the questionnaire obtained in the translation process was then revised by a committee of experts, consisting of physicians from several services in the four hospitals (15 from MCH, 15 from BCH, 10 from NCH and 3 from XXPH). The committee revised the pre-final survey regarding the adequacy and relevance of its content and clarity of questions. The expert committee approved unanimously the pre-final version of the questionnaire, which was then considered as MPCKAS final version. However, the committee recommended that participants should be clarified regarding the meaning of “hospice” (“*hospício*” in Portuguese) since the common meaning of that word in Portuguese speaking countries is a medical institution where specialized care is provided to patients with mental disorders, and not an institution that focuses on the palliative support of terminally ill patients.

The MPCKAS assesses knowledge, attitudes and practices of physicians covering issues related to palliative care, and includes six categories: 1) demographic; 2) concept and philosophy of palliative care; 3) disclosure of diagnosis and breaking bad news; 4) therapeutic decision issues; 5) end-of-life decision making issues and 6) euthanasia.

Physicians who consented to participate in the study were included. Unanswered surveys were excluded. Descriptive statistics were used as appropriate: absolute and relative frequencies for categorical variables and medians with 95% confidence intervals (95% CI) for age and clinical practice, in years. To evaluate independence between categorical variables, chi-squared test for independence was used. Normality of quantitative variables was verified by visual analysis of histograms and confirmed by Kolmogorov-Smirnov test. For mean comparison of normally distributed quantitative variables, Student t-test was performed. In the case of non-normally distributed quantitate variables, the nonparametric Mann-Whitney test was used. All the information collected was analyzed with SPSS software (v. 25). In all statistical tests, p-values were considered significant if less or equal to 0.05.

The study was approved by the Institutional Committee of Bioethics for Health of the Faculty of Medicine & Maputo Central Hospital with number CIBS FM&HCM/08/2018 and by the Bioethics Committee of the Faculty of Medicine of the University of Porto.

## Results

### 1. General characteristics and professional category

Two hundred and seven of the 306 distributed surveys were fully answered and therefore analyzed, resulting in response rates of 69.1% in Maputo Central Hospital (MCH), 90.6% in Beira Central Hospital (BCH), 64.9% in Nampula Central Hospital (NCH) and 75% in Xai-Xai Provincial Hospital (XXPH), conducting to an overall response rate of 67.6%. Respondents had a median age of 38 years and 54.6% were male. Thirteen percent were general practitioners, 40.1% specialists, and 46.9% residents (Table 1).

**Table 1:** Physicians’ demographic and professional characteristics (N = 207).

Variables	
Age in years, median [95% CI]	38 (36–40)
Age groups in years, n (%)	
25–34	76 (36.7)
35–44	69 (33.3)
45–54	45 (21.7)
55–65	17 (8.2)
Practise years, median [95% CI]	9 (8–11)
Practise years groups, n (%)	
0–13	138 (66.7)
14–27	48 (23.2)
28–41	21 (10.1)
Professional category, n (%)	
General Clinic	27 (13.0)
Resident	103 (49.8)
Specialist	77 (37.2)
Sex, n (%)	
Male	113 (54.6)
Female	94 (45.4)
Main specialties*, n (%)	
Surgery	44 (26.1)
Pediatrics	26 (15.8)
Gynecology	24 (14.5)
Medicine	23 (13.9)

*Only 165 professionals gave information.

### 2. General knowledge and attitudes in palliative care (Q1-Q9)

The percentage of correct answers referring to general knowledge and attitude in palliative care ranged between 55.7% and 94.1%. Only 25% of the participants correctly answered all five questions (Q1-Q5, see Table 2 for more detailed information).

**Table 2:** General palliative care knowledge and attitudes.

Q1-Q6 questions	n (%)
^a^Q1. PC should be provided for patients for whom there is not another alternative treatment (N = 206)	164 (79.6)
^a^Q2. PC should be done without any other ongoing treatment (N = 204)	153 (75.0)
^a^Q3. PC has different meanings for each individual (N = 202)	190 (94.1)
^a^Q4. Accepting death is a requirement to participate in PC (N = 203)	113 (55.7)
^a^Q5. There is no difference between PC and hospice (N = 200)	144 (72.0)
Participants who answered correctly Q1 to Q5. (N = 192)	48 (25.0)
^b^Q6. When should cancer patients receive PC? (N = 204)	
When cancer is first diagnosed	30 (14.7)
When cancer treatment is no longer helpful	171 (83.8)
With uncontrolled symptoms	83 (40.7)
When patients are psychologically disturbed	20 (9.8)
When patients ask for PC	56 (27.5)
When survival is expected to be for less than 3 months	37 (18.1)
When survival is expected to be for less than 6 months	38 (18.6)
Others	10 (4.9)

^a^ Respondents could respond with True, False, or Unknown. n (%) indicate correct answer frequencies.

^b^ Respondents could select more than one item in response (thus, percentages add up to more than 100%).

Two main items were considered when referring oncology patients to palliative care: when patients could not undertake surgery, radiotherapy, chemotherapy and other anti-cancer therapies (83.8%) and when the patients' symptoms could no longer be adequately controlled (40.7%). Only 14.7% of the participants believed that referral to palliative care should be made when cancer is first diagnosed, although 87.3% believed that early palliative care integration can improve patient’s quality of life (Q9).

### 3. Diagnostic disclosure and breaking bad news (Q10-Q15)

Most of the participants (61.5%) believed they should inform patients of an unfavorable prognosis although the great majority (99.5%) believed that it is the doctor’s responsibility to disclose diagnosis to patients. However, more than half (60.1%) preferred to disclose it to family members first (Table 3). The reasons invoked for rare or no disclosure of cancer diagnosis were (N = 39): negative psychological results (43.6%), difficulties in discussing the diagnosis (12.8%), provider is not prepared to deal with the situation (12.8%), lack of time (10.3%), the information is irrelevant to the patient (7.7%), and other reasons (12.9%). Most of the participants (88.2%) reported that their patients/caregivers often need more information regarding the disease. The most frequent reasons mentioned by physicians as to why patients/caregivers do not need more information about their illnesses were (N = 16): they understand the information they received (56.3%), they are afraid to ask questions (31.3%), and they think patients have difficulty understanding medical language (12.5%).

**Table 3:** Disease information disclosure and breaking bad news.

Q10-Q15 questions	n (%)
Q10. Is it important to disclose an unfavorable prognosis to patients? (N = 205)	
Yes	126 (61.5)
No	8 (3.9)
If the patient requests disclosure	4 (2.0)
If the family/caregivers request disclosure	19 (9.3)
It depends on situations^a^	48 (23.4)
Q11. Must the health care provider reveal the cancer diagnosis to patient/caregiver? (N = 205)	
Yes	149 (72.7)
No	19 (9.3)
Rarely	20 (9.8)
Only at patient request	17 (8.3)
Q12. Are patients/caregivers satisfied with the information you currently provide? (N = 202)	
Yes	116 (57.4)
No	86 (42.6)

a “It depends on the situation”- means decisions are on a case-by-case basis, considering the physical and psychological conditions, religion, and the cultural background of individuals.

### 4. Treatment decisions (Q16-Q17)

In the fourth part of the questionnaire, 85.8% of the physicians believed that patients must give an opinion about their treatment decision, and the most frequent reasons were to improve compliance of the patient to the treatment (76.5%) and so patients could be aware of side effects (46.3%). Some of the respondents who preferred not to involve patients on treatment decisions thought that it is difficult for them to discuss treatment with patients (30%) or they do not have enough time to discuss treatments with patients (30%). The majority of participants agreed that family should contribute in discussions regarding treatment options for the patient (92.6%).

### 5. Decision making and end-of-life issues (Q18-Q24)

Only 15.2% of the participants knew what advanced directives are (Q18), 50% knew what an order of “do-not-resuscitate” (Q19) is, and 43.3% choose to consider patient’s wish when he/she prefers to forgot life sustaining treatments (Q20). As described in Table 4, 19.3% thought that terminally ill cancer patients should receive cardiopulmonary resuscitation.

**Table 4:** Decision making and end-of-life issues.

Q21-Q24 questions	n (%)
Q21. Do you approve using CPR for terminally ill cancer patients? (N = 202)	
Yes	39 (19.3)
No	94 (46.5)
It depends on the situation^a^	69 (34.2)
^b^Q22. What factors do you believe will affect a patient and family’s decision? (N = 201)	
Disease prognosis	163 (81.1)
Symptom burden	105 (52.2)
Other disease and comorbidities	114 (56.7)
Religious beliefs	136 (67.7)
Economic status	125 (62.2)
The patient’s own wishes/preferences	104 (51.7)
Past experiences with death	105 (52.2)
All selected	80 (39.8)
Q23. Should a conflict arise between the patient’s wishes and the family’s wishes in the decision-making process, who would you support? (N = 199)	
Patients	66 (33.2)
Family	11 (5.5)
It depends on the situation^a^	122 (61.3)
Q24. If the patient is no longer competent and the family’s wishes conflict with those previous expressed by the patient, who would you support? (N = 202)	
Patients	39 (19.3)
Family	45 (22.3)
It depends on the situation^a^	118 (58.4)

a”It depends on the situation” means to decide on a case-by-case basis, considering the physical and psychological conditions, religion, and the cultural background of individuals.

^b^ Respondents could select more than one item in response (thus, percentages add up to more than 100%).

### 6. Euthanasia and related issues (Q25-29)

Most participants (89.4%) reported being familiar with euthanasia, 66.7% knew what “active euthanasia” is, 65.3% knew what “passive euthanasia” is, 38.8% knew what “physician-assisted suicide” is, and 51.3% knew what "palliative sedation” is. Overall, only 24.0% reported being familiar with all five euthanasia concepts. The years of practice distribution between the physicians familiar with all concepts was not found to be significantly different from the physicians not familiar with all concepts (both medians equal to 9 years; U = 3354; W = 14529; p = 0.663).

## Discussion

### General knowledge and attitudes in palliative care

This was the first survey of palliative care knowledge and attitudes in Mozambique, surveying the main hospitals in the main regions of the country and the only provincial hospital with a standalone isolated provision of palliative care service. Although Xai-Xai is the only provincial hospital in the country with this service and had the highest percentage of correct answers, this difference was not considered significant. There was also no significant difference in the years of practice between physicians who correctly answered all five palliative care knowledge questions.

From a public health point of view and because Xai-Xai has a standalone palliative care service for more than 5 years, significant differences in knowledge, attitudes and practices were expected for these health professionals. There is a need in palliative care advocacy not only in Xai-Xai but in all hospitals with these service in Gaza Province. And the fact that no significant differences re found between the years of practice of those who responded correctly and those who did not, calls attention to the need to introduce the palliative care curricula for health students at all levels and training for health professionals regardless of years of practice.

The results we obtained revealed that only 14.7% of the participants recommend palliative care when cancer is first diagnosed, which is lower than the 32.6% reported in a Chinese study [6]. Most professionals had the opinion that referral to palliative care should be done when patients can no longer undergo other anti-cancer therapies, which is similar to other countries [7]. However, this practice may have a negative impact on quality of life for some patients, because they are referred to palliative care at an advanced stage of disease. In our research, 87.3% believed that early palliative care integration could improve patients' quality of life, which is higher than the 72.5% reported for Chinese physicians [6], but less than 1/3 would recommend palliative care for cancer patients when patients attended the clinic for the first time.

The World Health Organization adopted a resolution to strengthen palliative care as a component of comprehensive lifelong care. This resolution emphasized the need to integrate palliative care into each country's health system to ensure access to all those in need, and also to provide mandatory palliative care training and knowledge for health professionals. Palliative care should be introduced as early as possible in patients with severe illness, providing early guidance for symptom control, with the aim of improving quality of life, care satisfaction and reduction of costs related to the hospital course of these patients [9,10]. This can be achieved through advanced care planning and individual care goals considering values ​​and preferences [11]. Some countries have developed legislation requiring palliative care to be provided at the time of diagnosis [12]. Over the years, these countries have adopted educational programs in order to improve doctors' knowledge so as to overcome their difficulties in this approach, and are already reporting positive results [13].

### Diagnostic disclosure and breaking bad news

Poor communication is one of the most common barriers to the provision of quality palliative care [12]. Disclosure of cancer diagnosis to a patient or caregiver is one of the most challenging communication tasks for physicians, considering that 25–35% of newly diagnosed patients present high levels of emotional distress [14]. Although interpersonal and communication skills are a core competency during the training of residents, this topic may not be a priority in surgical specialties [15]. Revealing a diagnosis of cancer is an art as is discussing the treatment options that follow. The discussion should be tailored to the individual with the involvement of family members, adapted to different patient needs [16]. The offer of physical treatment is considered the easiest discussion. However, disclosure of diagnosis and discussion of end-of-life issues are more challenging and uncomfortable [17]. Specific training on this is essential for quality palliative care.

When the patient has the capacity to understand and discernment to make decisions regarding the proposed treatment, it should be him/her, and only he/she, who consents. However, patients do not always have this capacity, and providers have to deal with incapacity situations, both in adults and minors [18]. In our study, the family-centered model of communication prevailed because most of the physicians preferred to first disclose diagnosis to family members rather than patients. Similar to our study, a Portuguese study described that some professionals prefer not to involve patients in treatment decisions, because they think it can negatively affect the patient psychologically [7]. However, when not involving the patient we are breaking the principle of patient autonomy.

### Treatment decision and end-of-life issues

Studies have shown an increase in aggressive care such as chemotherapy in the last month of life, particularly in cases where there is a lack of confrontation of end-of-life issues between doctors, patients, and caregivers. This can lead to a lower patient satisfaction, psychological distress, and poorer quality of life at the end of life [19]. In our study, there are some physicians who do not involve patients in the treatment decisions because it is difficult for them to discuss treatment with the patients. Clear consensus is lacking regarding retention or withdrawal of life-sustaining treatment, such as cardiopulmonary resuscitation, ventilatory support, artificial hydration, nutrition, sedative drugs used at the end-of-life, and terminology of euthanasia. Dr. Dame Cicely Saunders, founder of the modern palliative care movement, stated: "How people die is in the memory of those who live." Without a clear understanding of the ethical principles and policies that guide the management plans at the end-of-life, such care can lead to conflicts and dilemmas among health professionals [20].

Advance directives serves as a legal document that allows competent patients to give instructions on the health care they would like to receive when they are no longer competent to make their own decisions [21]. Not all countries have implemented these policies in their health system. Advance directives are most discussed in very high income countries such as the USA, Canada, United Kingdom, Netherlands, and Switzerland [22–24]. Mozambique has no laws relating to end-of-life issues, and ethical principles of good medical practice prevail. Most participants evaluate individual cases before making a decision on questions related to cardiopulmonary resuscitation in situations where the patient's desires conflict with the wishes of the family, especially when the patient is no longer competent.

The decision to suspend or withdraw medical interventions ideally should be made with the family, to ensure that they understand the underlying reason for such decisions. In case of withdrawal refusal from the family, doctors should refer the case to a colleague for a second opinion and it is up to the latter to agree to continue treatment or to negotiate more with the family. Guiding principles at the end-of-life include autonomy and beneficence. However, patients have the right to refuse medical interventions even though they may be beneficial. The problem arises in the absence of advance directives and when the patient is no longer competent, resorting in this way to a legal representative. Because these are usually close relatives, they may make emotional decisions that represent their own preferences rather than the patient’s [25].

One of the most common fears health professionals face is the use of end-of-life sedatives, since they associate this act with an acceleration of death due to hypotension and respiratory depression in debilitated patients. As a result, inadequate pain relief and sedation for terminal patients is common. In our sample, half of physicians claimed to know what palliative sedation is, but we believe not all of them use it in daily practice. Evidence has shown that if morphine and benzodiazepines are used properly to relieve end-of-life symptoms, they do not impact patient survival [26,27].

### Euthanasia and related issues

In this study, although few physicians were familiar with all concepts related to euthanasia (24%), the number is higher than the 14.5% reported in a Chinese study [6]. Interestingly, the years of practice between the physicians familiar with all concepts was not significantly different from the physicians not familiar with all concepts related to euthanasia.

### Limitations

Because the instrument was self-completed, not all questions were answered in all surveys. Only 4 hospitals were surveyed, so these results may not be generalizable to the whole country.

## Conclusions

Doctors of the three main hospitals of Mozambique and the only provincial hospital with a standalone palliative care service, have cursory knowledge about palliative care. Paternalism and the family-centered model are the most prevalent. Decision making is based on medical ethical principles considering each individual case, especially when the patient is no longer competent. Further research and palliative care education in the country is urgently needed to improve palliative care knowledge and practice in Mozambique.
